# Upregulation of miR-17-3p is associated with HbF in patients with β-thalassemia and induces γ-globin expression by targeting BCL11A

**DOI:** 10.1186/s13023-025-03806-0

**Published:** 2025-05-30

**Authors:** Siwen Zhang, Meihuan Chen, Wantong Zhao, Junhao Zheng, Yanhong Zhang, Aixiang Lv, Jingmin Li, Hua Cao, Liangpu Xu, Hailong Huang

**Affiliations:** 1 Medical Genetic Diagnosis and Therapy Center of Fujian Maternity and Child Health Hospital, College of Clinical Medicine for Obstetrics & Gynecology and Pediatrics, Fujian Medical University, Fujian Provincial Key Laboratory of Prenatal Diagnosis and Birth Defect, No. 18 Daoshan Road, Gulou District, Fuzhou, Fujian, 350001 China; 2 The School of Medical Technology and Engineering, Fujian Medical University, Fuzhou, China; 3 Yanjiang District People’s Hospital of Ziyang City, Ziyang, Sichuan China; 4 Fujian Clinical Research Center for Maternal-Fetal Medicine, Fuzhou, China; 5 National Key Obstetric Clinical Specialty Construction Institution of China, Fuzhou, China

**Keywords:** β-thalassemia, miR-17-3p, BCL11A, HbF, Erythropoiesis

## Abstract

**Background:**

Large number of microRNAs (miRNAs) have been found to be dysregulated in β-thalassemia, but their roles in β-thalassemia are poorly reported. This study aims to investigate the clinical significance of miR-17-3p in β-thalassemia, and to elucidate its regulatory effect on erythropoiesis and γ-globin expression.

**Methods:**

We collected peripheral blood samples from 17 patients with β-thalassemia (including intermedia and major subtypes) and 17 healthy controls, and the expression levels of miR-17-3p, BCL11 transcription factor A (BCL11A) and γ-globin were detected by qRT-PCR, and their correlations were analyzed. The regulation of miR-17-3p on BCL11A was evaluated in K562 cells by bioinformatics, luciferase reporter gene assay, fluorescence in situ hybridization and Western blotting. Furthermore, the effects on miR-17-3p overexpression and knockdown on erythropoiesis including cell proliferation, cell cycle, cell apoptosis, and erythroid differentiation of K562 cells were assessed by CCK-8, flow cytometry and benzidine blue staining.

**Results:**

The expression of miR-17-3p was upregulated in patients with β-thalassemia, and was positively correlated with fetal hemoglobin (HbF) levels. BCL11A expression was reduced in β-thalassemia patients, and was negatively correlated with miR-17-3p and γ-globin expression. BCL11A was identified as a target gene of miR-17-3p, and was negatively regulated by miR-17-3p. Furthermore, miR-17-3p mediated the upregulation of γ-globin expression in K562 cells through BCL11A. In addition, neither overexpression nor knockdown of miR-17-3p appeared to affect cell proliferation, cell cycle, cell apoptosis or erythroid differentiation of K562 cells in vitro.

**Conclusion:**

The upregulated miR-17-3p is associated with HbF in patients with β-thalassemia. Although miR-17-3p does not affect erythropoiesis, it promotes γ-globin expression by targeting BCL11A, suggesting that miR-17-3p may be a promising miRNA for the treatment of β-thalassemia.

**Supplementary Information:**

The online version contains supplementary material available at 10.1186/s13023-025-03806-0.

## Highlights

miR-17-3p expression is upregulated in patients with β-thalassemia, and is positively correlated with HbF.

miR-17-3p shows no significant effect on cell proliferation, cell cycle, apoptosis and erythroid differentiation, suggesting it may not affect erythropoiesis.

BCL11A is a target gene of miR-17-3p, and miR-17-3p induces γ-globin expression by downregulating BCL11A.

**Supplementary Information:**

The online version contains supplementary material available at 10.1186/s13023-025-03806-0.

## Introduction

Thalassemia is a group of hereditary blood disorders that can be fatal and disabling, posing a serious threat to human health [1]. Depending on the different globin peptide chains affected, thalassemia can be categorized into different types such as α, β, γ, δ, δβ, and εγδβ, among which α-thalassemia and β-thalassemia are the most prevalent. In mainland China, the incidence of α-thalassemia and β-thalassemia is respectively 2.95% and 0.67%, and highly prevalent in Southern China, especially in the regions of Guangdong, Guangxi, Hainan, and Fujian, with over 200 million individuals affected [2]. β-thalassemia is primarily caused by mutations in the β-globin gene located on the human chromosome 11p15.4, resulting in reduced or deficient synthesis of β-globin chains, causing an imbalance between α-globin chains and β-globin chains. The accumulation of excess α-globin chains on the erythrocyte membrane eventually leads to ineffective erythropoiesis and hemolytic anemia [3].

According to the severity of anemia, β-thalassemia can be classified as minor, intermedia, and major, and symptoms in patients with intermedia and major progressively worsen after birth. At present, β-thalassemia is more commonly classified into non-transfusion-dependent (NTD) and transfusion-dependent (TD) based on whether it is transfusion-dependent or not. The treatment of β-thalassemia currently relies on blood transfusion, chelation therapy, or bone marrow transplantation [4], but these measures still impose a heavy financial burden on patients. At present, gene therapy has become a promising therapeutic method. Numerous studies have demonstrated that fetal hemoglobin (HbF) induction therapy is an effective treatment for β-thalassemia patients, although the majority of these studies lack the requisite clinical trial data for verification [5].

During development, the transformation of the main component of human hemoglobin from HbF to adult hemoglobin is influenced by multiple transcription factors [6], such as BCL11 transcription factor A (BCL11A), Krüppel like factors (KLFs), and MYB. BCL11A is predominantly expressed in brain and hematopoietic tissues and is regulated by various microRNAs (miRNAs), transcription factors, and genetic variations [7]. The transcription factor BCL11A can preferentially binds to the distal motif and destroy the distal motif by binding to the promoter region of the γ-globin gene, thus inhibiting the expression of γ-globin [8]. Numerous studies have confirmed that inhibition of BCL11A expression can induce the activation of endogenous γ-globin gene and raise the content of HbF levels, thereby ameliorating the clinical manifestations in individuals with β-thalassemia [6, 8–10]. For instance, the CRISPR-Cas9-based BCL11A enhancer-editing strategy can maintain significant elevations in HbF levels, which are substantial enough to alleviate the severity of transfusion-dependent thalassemia [11].

MiRNAs are a class of endogenous non-coding RNA molecules of approximately 19–25 nt in length that are critical regulators of about 40–70% of genes in the human genome [12]. In β-thalassemia, certain miRNAs bind to erythroid-specific transcription factors, thereby modulating γ-globin expression and influencing the development and maturation of erythroid cells, consequently impacting the clinical manifestations of β-thalassemia. For instance, the direct targeting of KLF1’s 3’-UTR by miR-326 leads to down-regulation of KLF1, consequently resulting in decreased expression of BCL11A and increased expression of the γ-globin gene [13]. miR-92a-3p could up-regulate γ-globin level and attenuate oxidative stress and apoptosis in erythroid precursor cells through the downregulation of BCL11A [14].

In a previous study [15], our laboratory conducted high-throughput miRNA sequencing on peripheral blood samples from β-thalassemia patients (GSE241765), and found 196 differentially expressed miRNAs including miR-17-5p (downregulation at fold change of 0.26) and miR-17-3p (downregulation at fold change of 0.33). As a member of the miR-17-92 cluster, previous studies focused on the roles of miR-17-3p in myocardial ischemia-reperfusion injury and tumorigenesis [16–17]. We predicted that miR-17-3p may have a regulatory effect on BCL11A by bioinformatics. However, the role of miR-17-3p in β-thalassemia remains unclear. Therefore, the current study aims to further examine the expression of miR-17-3p in patients with β-thalassemia, analyze the clinical value, and investigate its regulatory effects on erythropoiesis and γ-globin expression through in vitro functional assays. Our study will deepen the understanding of miR-17-3p’s role in β-thalassemia development.

## Materials and methods

### Subjects

Seventeen healthy controls (8 males and 9 females with an average age of 8.59 ± 2.43 years) and seventeen patients with β-thalassemia (10 males and 7 females with an average age of 9.47 ± 2.03 years) who were screened out by high-throughput gene sequencing were collected at Fujian Maternity and Child Health Hospital. Based on genotypes and clinical symptoms (anemia, hepatomegaly, splenomegaly, and delayed growth and development), these patients were classified into β-thalassemia intermedia and β-thalassemia major (Supplementary Table 1). Inclusion criteria for the healthy control: peripheral blood hemoglobin (Hb) > 120 g/L, MCV ≥ 80 fL, MCH ≥ 27 pg, no pathogenic mutation in the β-globin gene by genetic diagnosis; exclusion criteria: the presence of other underlying diseases. Inclusion criteria for the β-thalassemia: Hb < 90 g/L, diagnosed with β-thalassemia by genetic diagnosis; exclusion criteria: the presence of other underlying diseases, receiving blood transfusion therapy within the last month. This study obtained written informed consent from all participants (or their guardians) and approval of the Ethics Committee of Fujian Maternity and Child Health Hospital (No. 073, 2019). The study was conducted in strict compliance with the Declaration of Helsinki (as revised in 2013).

### Samples collection and clinical indicators

5 ml of peripheral blood was collected from each subjects, and RNA samples were isolated and purified from whole blood cells using the PAXgene Blood RNA Kit (Qiagen, Hilden, Germany) and the RNA samples were stored at -80 °C for further experimental analysis. Simultaneously, peripheral blood was collected in tubes containing Ethylenediaminetetraacetic Acid (EDTA) anticoagulant for hematological parameters and hemoglobin component analysis, and in tubes containing Heparin anticoagulant for biochemical indicator analysis. Blood cell parameters including red blood cell (RBC), hemoglobin (Hb), mean corpuscular volume (MCV), mean corpuscular hemoglobin (MCH) and platelet (PLT) were analyzed on the Sysmex XN-3000 automatic blood analyzer (Sysmex; Shanghai, China). The hemoglobin composition and levels, such as hemoglobin A (HbA), hemoglobin A2 (HbA2) and fetal hemoglobin (HbF) were analyzed on the automatic capillary electrophoresis system (CapillaryS 2, software version 6.2; Sebia, Paris, France), and biochemical indicators containing total bile acid (TBA), total bilirubin (TBIL), direct bilirubin (DBIL), alanine amiotransferase (ALT), aspartate aminotransferase (AST), alkaline phosphatase (ALP), γ-glutamyl transpeptidase (GGT), total protein (TP), albumin (ALB), globulin (GLOB), prealbumin (PA), cholinesterase (CHE), serum ferritin (SF) were measured using chemiluminescent particle immunoassay (CMIA) (Abbott; ARCHITECT ci16200, USA).

### Cell culture and cell transfection

Human chronic myeloid leukemia cell line K562 was purchased from Shanghai Anwei Biotechnology Co, LTD (Shanghai, China). Human embryonic kidney cell line 293T (HEK-293T) was stored at the Medical Research Center of Fujian Maternal and Child Health Hospital. K562 cells were cultured in Roswell Park Memorial Institute (RPMI) 1640 medium (Gibco, USA) supplemented with 10% fetal bovine serum (BI, Australia) and 1% Penicillin–Streptomycin (Gibco, USA). HEK-293T cells were cultured in Dulbecco’s modified Eagle medium (Gibco, USA) supplemented with 10% fetal bovine serum and 1% Penicillin–Streptomycin.

The miR-17-3p mimics (5’-ACUGCAGUGAAGGCACUUGUAG-3’) and inhibitors (5’-CUACAAGUGCCUUCACUGCAGU-3’), and negative control mimics (NC mimics, 5’-UUCUCCGAACGUGUCACGUTT-3’) and negative control inhibitors (NC inhibitors, 5’- CAGUACUUUUGUGUAGUACAA-3’) were purchased from Genepharma (Genepharma Inc., Shanghai, China). Cell transfection was performed with these oligonucleotides at a concentration of 100 nM using Cell Line Nucleofector™ Kit V Solution (Lonza, Switzerland) according to the manufacturer’s protocol. After transfection, cells were cultured in a humidified atmosphere (37 °C with 5% CO_2_) for 48 h, as the manufacturer’s instructions.

### RNA extraction and quantitative real-time polymerase chain reaction (qRT-PCR)

Total RNA Extraction Kit (Promega, Shanghai, China) and PAXgene blood RNA kit (Qigen, Germany) were used to extract total RNA from cultured cells and peripheral blood samples according to the manufacturer’s instructions, after which the RNA samples were stored at -80 °C. The concentration and purity of the RNA samples were assessed using 260/280 nm and 260/230 nm ratios with the biometric MULTISKAN GO (Thermo, USA).

The cDNA of mRNAs was synthesized using a PrimeScript™ RT-PCR kit (Takara Bio, Inc., Japan) according to the manufacturer’s instructions. The cDNA of miRNAs was synthesized using a Mir-X TM First Strand Synthesis kit (Takara Bio, Inc., Japan) according to the manufacturer’s instructions. The PrimeScript™ RT-PCR kit and Mir-X miRNA qRT-PCR TB Green^®^ Kit (Takara, Japan) were used for qRT-PCR. RT-PCR was performed on the QuantStudio 3 and 5 Real-Time PCR Systems (Applied Biosystems, USA). The miRNA levels were normalized to small nuclear U6, and the mRNA levels were normalized by GAPDH. The following primers were used for qRT-PCR: miR-17-3p sense: 5’-CCACTGCAGTGAAGGCACTTGTAG-3’ and antisense supplied by the kit; U6 sense: 5’-CTCGCTTCGGCAGCACA-3’ and antisense: 5’-AACGCTTCACGAATTTGCGT’; BCL11A sense: 5’-ACAGGAACACATAGCAGATAAAC-3’ and antisense: 5’-TATTCTGCACTCATCCCAGG-3’; γ-globin sense: 5’-CTGGGAAGGCTCCTGGTTG-3’ and antisense: 5’-CAGAGGCAGAGGACAGGTTG-3’; GAPDH sense: 5’-CAACAGCGACACCCACTCCT-3’ and antisense: 5’-CACCCTGTTGCTGTAGCCAAA-3’. Relative expression levels of miR-17-3p, BCL11A, and γ-globin were calculated using the comparative cycle threshold method.

### Protein extraction and western blotting analysis

Total protein was obtained from cells by RIPA lysis buffer (Beyotime Biotechnology, Shanghai, China). Quantification of proteins was performed using a BCA protein concentration assay kit (Beyotime Biotechnology, Shanghai, China). The denatured protein was subjected to sodium dodecyl sulfate-polyacrylamide gel electrophoresis (SDS-PAGE) and transferred onto polyvinylidene difluoride (PVDF) membranes (Millipore, USA) in an ice bath at 300 mA for 90 min. After blocking with 5% skim milk powder (Beyotime Biotechnology, Shanghai, China), the membranes were incubated with primary antibodies, including anti-BCL11A (1:1000, ab19487), anti-γ-globin (1:1000, ab156584) and anti-GAPDH(1:5000, ab8245) at 4℃ overnight. After rinsing three times with TBS/0.1% Tween 20 (TBST), the blots were probed with horseradish peroxidase-labeled goat anti-rabbit IgG secondary antibody (for γ-globin, 1:5000, ab205718) or horseradish peroxidase-labeled goat anti-mouse IgG secondary antibody (for GAPDH and BCL11A, 1:5000, ab205719) at 37 °C for 60 min, followed by visualization using Efficient chemiluminescence kit (Genview, USA), and measured with FluorChem M System (ProteinSimple, USA). All antibodies are sourced from Abcam (Abcam, Cambridge, Massachusetts, USA). The relative levels of each protein expression were determined by densitometric analysis using ImageJ software (Image J 1.51, NIH, USA).

### Cell proliferation assay

The CCK-8 kit (Dojindo, Japan) was applied to detect cell proliferation. Transfected K562 cells were seeded in a 96-well plate at a density of 1000 cells per well in 5% CO_2_ incubator at 37 °C for various times including 24 h, 48 h, 72 h and 96 h. Subsequently, 10 µL of CCK-8 solution was added to the corresponding wells of the plate and incubated for 2 h. The plate was gently mixed on a shaker before reading. The absorbance at 450 nm was measured using an enzyme immunoassay analyzer (Thermo Fisher Scientific, USA).

### Flow cytometry

Flow cytometry was used to detect the effects of miR-17-3p on cell cycle, apoptosis, and erythroid differentiation. For detecting cell cycle, 75% ethanol-fixed K562 cells were stained with 5 µL of propidium iodide (BD Biosciences, USA), and the cell cycle was detected by Flow cytometry (BD Biosciences, USA). The ratio of G0/G1, S, and G2/M phase cells was analyzed by Modfit software (Verity Software House, USA).

For detecting cell apoptosis, K562 cells were incubated with Annexin V-FITC/PI (BD Biosciences, USA) for 15 min according to the manufacturer’s instructions. Then apoptotic changes were analyzed using flow cytometry with FlowJo software (BD Biosciences, USA).

For detecting erythroid differentiation, Hemin (Beyotime Biotechnology, Shanghai, China) was added to the medium containing the transfected cells at a final concentration of 50 µM and after 96 h of incubation, erythroid differentiation was detected using the CD71/CD235a kit (BD Biosciences, USA). After 30 min incubation, cell differentiation changes were analyzed using flow cytometry and FlowJo software, and the proportion of CD71- and CD235a- positive cells was calculated.

### Benzidine blue staining

Hemoglobin was detected by benzidine blue staining during red lineage differentiation of K562 cells. The freshly prepared benzidine-H_2_O_2_ solution, containing 50 µL of 3% H_2_O_2_ per mL of 0.4% benzidine in 1% acetic acid, was added to the cell suspension and incubated for five minutes in the dark. Cells were then observed and images were taken under an inverted microscope (Nikon, Japan), and the positive rate was calculated as the proportion of blue cells among 100 cells counted in different fields.

### Fluorescence in situ hybridization

The miR-17-3p probe sequence (5’-CTACAAG + TGCCT + TCACTGCAGT-3’) was designed and synthesized by GenePharma, Shanghai, China. K562 cells were first fixed in 4% formaldehyde solution (Beyotime Biotechnology, Shanghai, China), and then incubated with 0.5% Triton (Beyotime Biotechnology, Shanghai, China). The FAM-labeled miR-17-3p probe was hybridized with the cells at 37 °C for 12 h. The nuclei of the cells were labeled with DAPI the following day. Cells were then photographed by the AX/AX R confocal microscope (Nikon, Japan).

### Dual luciferase reporter gene assay

Wild-type and mutant sequences of binding sites (wt-BCL11A and mut-BCL11A) were designed based on the predicted binding sites of miR-17-3p to BCL11A from miRanda (http://www.microrna.org), TargetScan (http://www.targetscan.org) and miRDB (http://mirdb.org), and the designed sequences were inserted into the luciferase reporter vector pmiR-RB-REPORT™. HEK-293T cells were seeded in 24-well plates at a concentration of 1 × 10^5^ cells per well. The miR-17-3p mimics, the NC mimics, the miR-17-3p inhibitors, and the NC inhibitors were cotransfected with the sequences inserted into the luciferase reporter vector pmiR-RB-REPORT™. 48 h later, luciferase activities were measured by the Lucifer Reporter Assay System (Promega, Shanghai, China). The ratio of Renilla luciferase activity to Firefly luciferase activity was considered as the relative luciferase activity (Firefly luciferase activity served as the internal control).

### Statistical analysis

All experiments were repeated independently three times. Statistical analyses were performed using SPSS version 26.0 (IBM SPSS Inc., USA) and GraphPad Prism software (GraphPad Software, USA). Measures that conformed to normal distribution were expressed as mean ± standard deviation and statistically compared between two groups using Student’s t-test and between multiple groups using one-way ANOVA. Measures that did not conform to normal distribution were expressed as median and quartiles M (P25, P75) and statistically compared using the Mann-Whitney U test. Bivariate correlations were analyzed using Pearson correlation analysis. The diagnostic value of miR-17-3p for β-thalassemia was analyzed using receiver operating characteristic (ROC) curves. The *P* values are indicated directly on the figure within the corresponding graphs; *P < 0.05, **P < 0.01 and ***P < 0.001 were considered statistically significant.

## Results

### Comparison of clinical indicators in patients with β-thalassemia and healthy controls

As shown in Supplementary Table 2, no statistically significant differences was found in the HbA2, ALP, GGT, ALB, and GLOB level between the two groups (*P* > 0.05). Compared to healthy controls, patients with β-thalassemia revealed significantly lower levels of RBC, Hb, MCV, MCH, HbA, TP, PA, and CHE (*P* < 0.05). Conversely, HbF, PLT, TBA, TBIL, DBIL, ALT, AST, and SF levels were significantly higher in patients with β-thalassemia (*P* < 0.05). These data confirmed the presence of anemia and impairment of liver function in patients with β-thalassemia.

### The relationship between miR-17-3p expression and clinical indicators in β-thalassemia patients

**Fig. 1:**
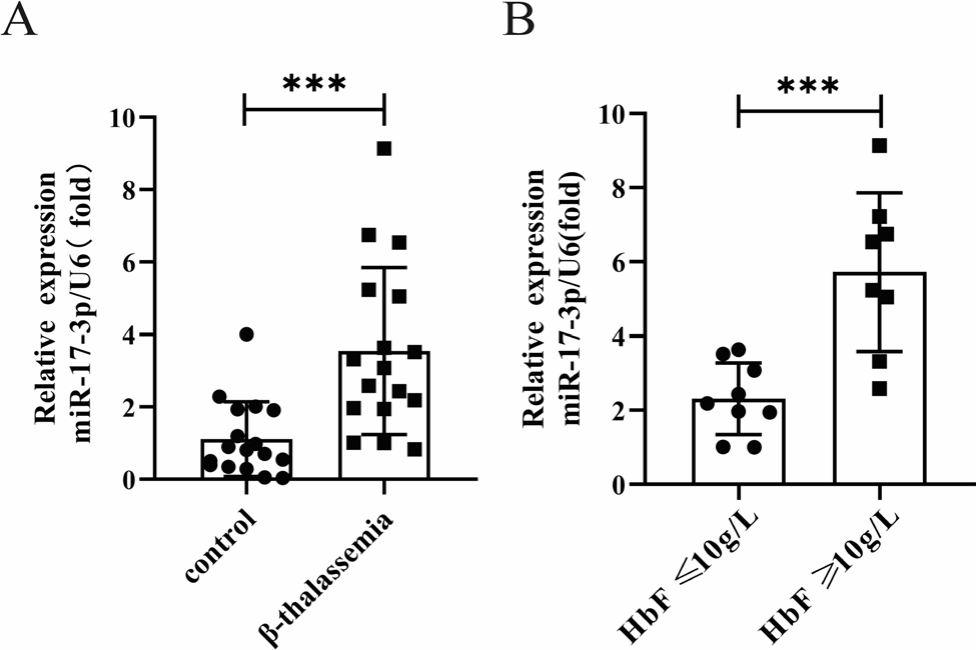
miR-17-3p expression in peripheral blood of patients with β-thalassemia. (**A**) qRT-PCR analysis of miR-17-3p expression in peripheral blood of patients with β-thalassemia and healthy controls. (**B**) The expression levels of miR-17-3p were evaluated in the HbF ≥ 10 g/L group and the HbF < 10 g/L group. qRT-PCR: quantitative real-time polymerase chain reaction, HbF: fetal hemoglobin. Error bars represented the means ± SD. ***, *P* < 0.001

RT-qPCR results showed that miR-17-3p expression levels were significantly higher in patients with β-thalassemia compared with healthy controls (3.54 ± 2.31 vs. 1.11 ± 1.03, *P* < 0.001) (Fig. 1A). Additionally, miR-17-3p expression levels in the patients with HbF ≥ 10 g/L were higher than those with HbF < 10 g/L (5.07 ± 2.40 vs. 2.89 ± 1.89, *P* < 0.001) (Fig. 1B).

To clarify the clinical significance of miR-17-3p in β-thalassemia, patients were divided into two groups based on the average level of miR-17-3p (3.54): high miR-17-3p group (*n* = 6) and low miR-17-3p group (*n* = 11), and the differences in these clinical indicators between the two groups were assessed. As shown in Table 1, compared to the low miR-17-3p group, levels of MCV, MCH, HbA, and ALP levels were significantly decreased in the high miR-17-3p group, while HbF, TBA, and GGT were significantly increased (all *P* < 0.05). Additionally, Pearson correlation analysis confirmed that miR-17-3p expression was negatively correlated with MCV (*r* = -0.733), MCH (*r* = -0.637), and HbA (*r* = -0.678), but positively correlated with HbF (*r* = 0.686), TBA (*r* = 0.643), and GGT (*r* = 0.713) (Table 2, all *P* < 0.05). These results demonstrated that miR-17-3p expression is upregulated in patients with β-thalassemia, and is correlated with increased levels of HbF and impaired liver function.

**Table 1:** The relationship between miR-17-3p expression and clinical indicators in β-thalassemia patients

Items	High miR-17-3p expression (*n* = 6)	Low miR-17-3p expression (*n* = 11)	t/Z	*P*
RBC (×10^12^/L)	3.85 ± 0.45	3.54 ± 0.41	-1.44	0.170
Hb (g/L)	82.50 (81.25,85.25)	79.00 (78.5,84.5)	-0.36	0.722
MCV (fL)	71.50 ± 4.51	80.27 ± 5.08	3.53	0.003
MCH (pg)	23.03 ± 1.32	25.64 ± 2.04	2.80	0.013
HbA (%)	70.09 (57.52,79.29)	92.33 (80.86,94.36)	3.02	0.009
HbA_2_ (%)	2.76 (2.45,3.12)	3.02 (2.96,3.35)	0.72	0.482
HbF (%)	27.12 (17.80,39.89)	4.73 (2.42,12.57)	-3.11	0.007
PLT (×10^9^/L)	411.67 ± 179.02	430.45 ± 211.78	0.18	0.857
TBA (µmol/L)	13.20 ± 4.85	6.45 ± 4.34	-2.95	0.010
TBIL (µmol/L)	28.50 ± 6.77	25.36 ± 9.51	-0.71	0.488
DBIL (µmol/L)	9.37 ± 4.08	9.38 ± 3.55	-0.19	0.854
ALT (U/L)	38.00 (25.25,43.25)	25.00 (14.6,43.5)	-0.17	0.864
AST (U/L)	31.00 (28.5,47.75)	28.30 (24.5,39.5)	-0.14	0.888
ALP (U/L)	182.00 ± 30.29	288.15 ± 143.92	2.35	0.037
GGT (U/L)	14.83 ± 1.94	11.97 ± 1.55	-3.34	0.004
TP (g/L)	70.17 ± 5.30	67.27 ± 4.95	-1.12	0.279
ALB (g/L)	44.27 ± 3.23	45.18 ± 1.54	0.08	0.435
GLOB (g/L)	26.13 ± 2.05	22.22 ± 4.23	-2.11	0.052
PA (g/L)	194.33 ± 31.77	200.64 ± 49.67	0.28	0.784
CHE (U/L)	5928.17 ± 815.00	7205.09 ± 255.77	1.18	0.258
SF (µg/L)	2882.83 ± 983.33	2849.76 ± 1172.44	-0.06	0.950

*Abbreviations*: RBC: red blood cell, Hb: hemoglobin, MCV: mean corpusular volume, MCH: mean corpuscular hemoglobin, HbA: hemoglobin A, HbA_2_: hemoglobin A2, HbF: fetal hemoglobin, PLT: platelet, TBA: total bile acid, TBIL: total bilirubin, DBIL: direct bilirubin, ALT: alanine amiotransferase, AST: aspartate aminotransferase, ALP: alkaline phosphatase, GGT: γ-glutamyl transpeptadase, TP: total protein, ALB: albumin, GLOB: globulin, PA: prealbumin, CHE: cholinesterase, SF: serum ferritin. Measures that conformed to a normal distribution were expressed as mean ± standard deviation, and t-test was used for statistical comparison between the two groups, while measures that did not conform to normal distribution were expressed as median and quartiles M (P25,P75), and Mann-Whitney U test was used for statistical comparison

**Table 2:** Pearson correlation analysis the correlations between miR-17-3p expression and clinical indicators in β-thalassemia patients

Items	*r*	*P*
MCV (fL)	-0.733	0.001
MCH (pg)	-0.637	0.006
HbA (%)	-0.678	0.003
HbF (%)	0.686	0.002
TBA (µmol/L)	0.643	0.005
ALP (U/L)	-0.293	0.253
GGT (U/L)	0.713	0.001

### The potential of miR-17-3p as a monitoring biomarker in patients with β-thalassemia

**Fig. 2:**
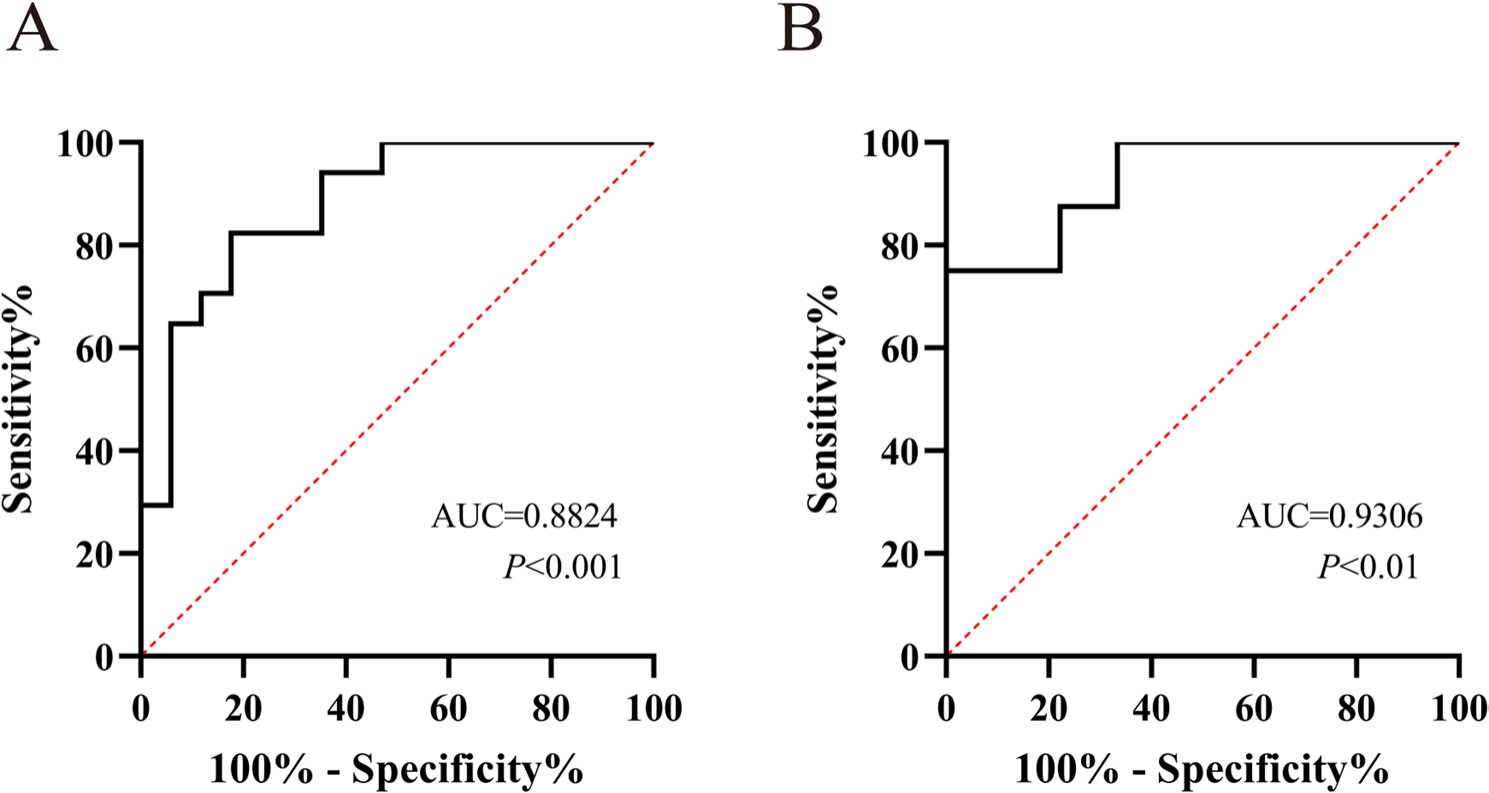
The potential of miR-17-3p as a monitoring biomarker for patients with β-thalassemia. (**A**) Distinguishing efficacy of miR-17-3p expression in β-thalassemia patients by ROC curve analysis. (**B**) Distinguishing efficacy of miR-17-3p in β-thalassemia patients with different levels of HbF. ROC: receiver operating characteristic, AUC: area under the curve

The ROC curves were used to examine the potential of miR-17-3 as a monitoring biomarker, and the area under the curve (AUC) of miR-17-3p in distinguishing patients with β-thalassemia from healthy controls was 0.8824, with 95% confidence interval (CI) of 0.77 to 1.00 and optimal diagnostic threshold of 1.942, corresponding to the sensitivity of 82.35% and the specificity of 82.35% (Fig. 2A, *P* < 0.001). We further analyzed the efficacy of miR-17-3p for the monitoring of β-thalassemia patients with different HbF levels, and the results showed that the cutoff value of miR-17-3p in distinguishing patients with HbF ≥ 10 g/L from those with HbF < 10 g/L was 4.339 (AUC = 0.9306, 95% CI: 0.07-1.0, sensitivity = 75.00%, specificity = 100.0%) (Fig. 2B, *P* < 0 0.001). These data demonstrated that miR-17-3p has a viable performance in determining β-thalassemia with different conditions, and might be used as a monitoring biomarker for this disease.

### Identification of BCL11A as a target gene of miR-17-3p

**Fig. 3:**
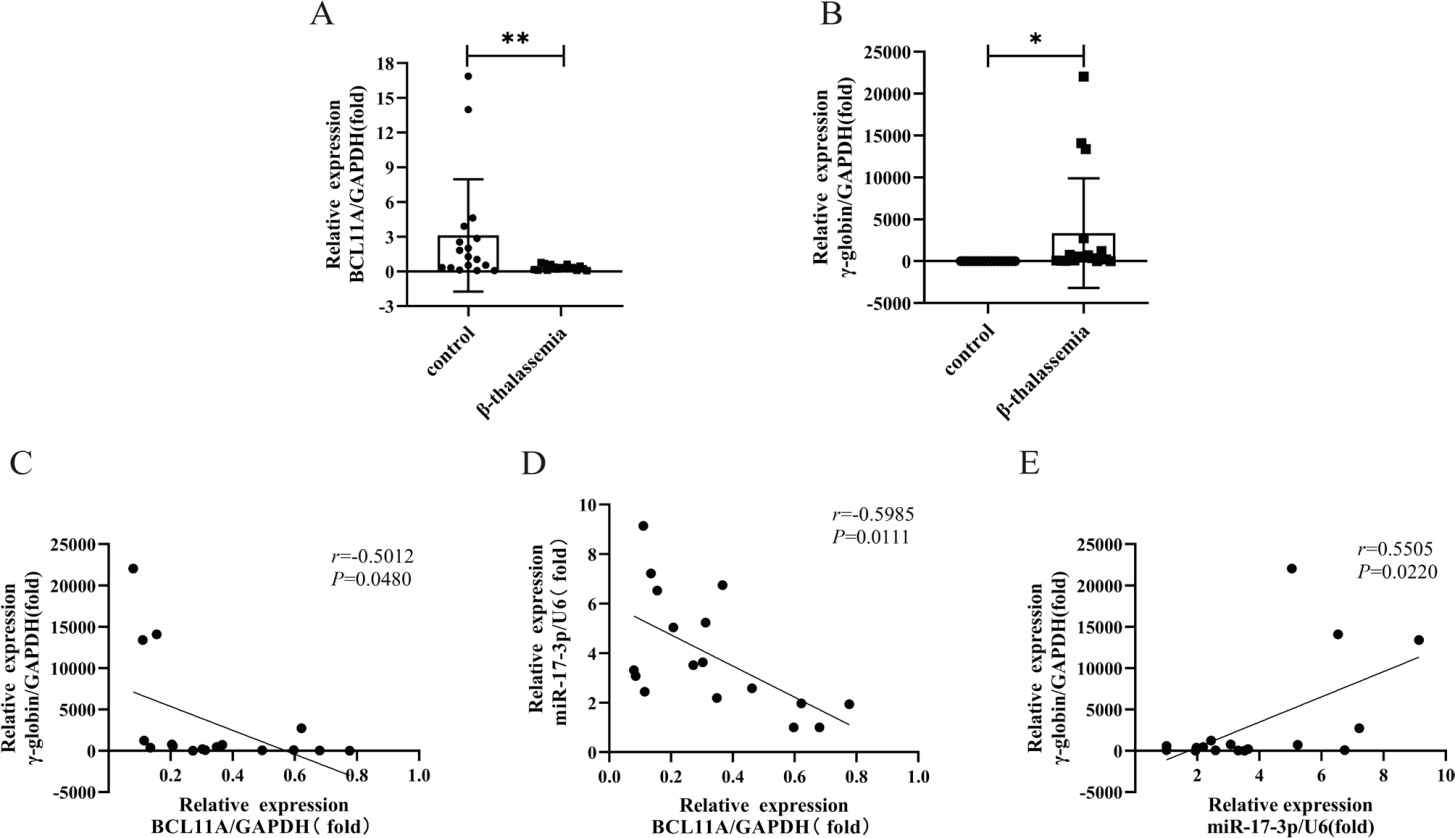
The expression of BCL11A in patients with β-thalassemia and its correlation with miR-17-3p and γ-globin. (**A**) Differential analysis of BCL11A mRNA expression in patients with β-thalassemia and healthy controls. (**B**) Differential analysis of γ-globin mRNA expression in patients with β-thalassemia and healthy controls. (**C**-**E**) Pearson correlation analysis of BCL11A, γ-globin and miR-17-3p expression in patients with β-thalassemia. BCL11A: BCL11 transcription factor A. Error bars represented the means ± SD. *, *P* < 0.05; **, *P* < 0.01

RT-qPCR results showed that BCL11A mRNA was lower in patients with β-thalassemia compared with healthy controls (Fig. 3A, *P* < 0.01). Additionally, γ-globin mRNA expression was increased in β-thalassemia patients compared to healthy controls (Fig. 3B, *P* < 0.05). Pearson correlation analysis indicated that BCL11A mRNA expression was negatively correlated with γ-globin (*r* = -0.5012) and miR-17-3p expression (*r* = -0.5985) (Fig. 3C and D, *P* < 0.05). However, a significantly positive correlation was found between miR-17-3p expression and γ-globin expression (*r* = 0.5505) (Fig. 3E, *P* < 0.05).

**Fig. 4:**
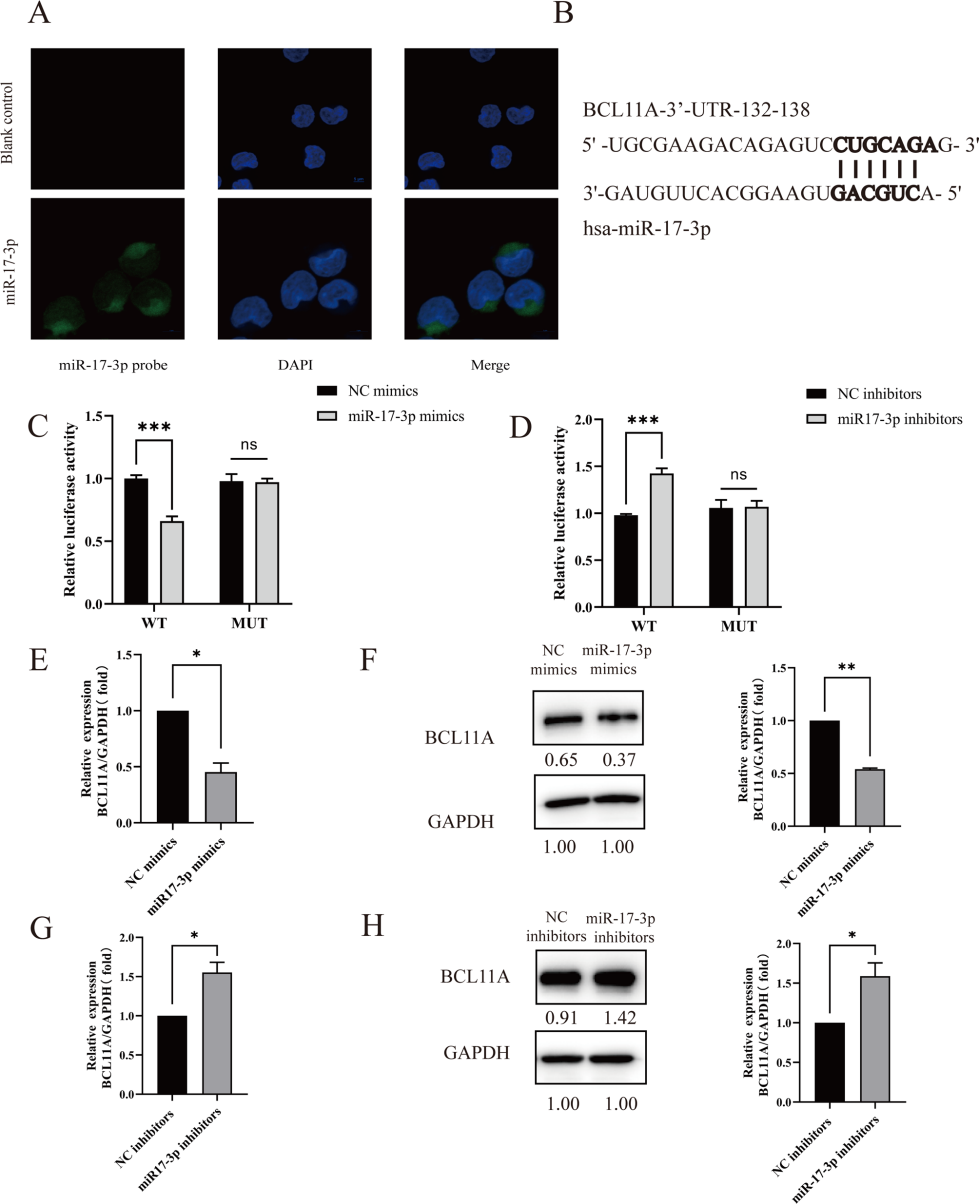
BCL11A as a target gene of miR-17-3p. (**A**) Fluorescent localization of miR-17-3p in K562 cells. (**B**) Extent of complementarity between miR-17-3p and binding sites present within BCL11A mRNA 3’-UTR. (**C**-**D**) Dual luciferase gene reporter assay of HEK-293T cells results show that miR-17-3p could bind to BCL11A mRNA 3’UTR. Expression analysis of BCL11A mRNA (**E**), and BCL11A protein (**F**) levels in the miR-17-3p mimics group and NC mimics group. Effect of knockdown of miR-17-3p on the expression analysis of BCL11A mRNA (**G**), and BCL11A protein (**H**) levels. Error bars represented the means ± SD. *, *P* < 0.05; **, *P* < 0.01; ***, *P* < 0.001; ns, *P* > 0.05

We then analyzed the sublocation of miR-17-3p in K562 cells, and fluorescence in situ hybridization showed that miR-17-3p was mainly localized in the cytoplasm (Fig. 4A). Bioinformatics analysis predicted that sites 132–138 of the BCL11A mRNA 3’-UTR were the binding sites for miR-17-3p (Fig. 4B). To explore the regulation of miR-17-3p on BCL11A, we first verified the transfection efficiency of miR-17-3p overexpression and knockdown in K562 and HEK-293T cells. RT-qPCR results showed that miR-17-3p expression level in K562 and HEK-293T cells was significantly higher in the miR-17-3p mimics group compared with the NC mimics group (Supplementary Fig. 1A, *P* < 0.001), while its expression was significantly lower in the miR-17-3p inhibitors group compared with the NC inhibitors group (Supplementary Fig. 1B, *P* < 0.001), indicating successful overexpression and knockdown of miR-17-3p in K562 and HEK-293T cells.

Then, we constructed a dual luciferase reporter assay in HEK-293T cells, and results showed a significant decrease in the relative luciferase activity of the wild-type BCL11A mRNA 3’-UTR vector in the miR-17-3p mimics group compared to the NC mimics group (Fig. 4C, *P* < 0.001). There was no statistically significant difference in the relative luciferase activity of the mutant BCL11A mRNA 3’-UTR vector between the miR-17-3p mimics group and the NC mimics group (*P* > 0.05). The luciferase activity of the wild-type but not the mutant BCL11A mRNA 3’-UTR vector was significantly promoted by the miR-17-3p inhibitors group (Fig. 4D, *P* < 0.001). In addition, by RT-qPCR and Western blotting analysis, we found that the mRNA and protein expression levels of BCL11A were decreased in K562 cells with miR-17-3p overexpression (Fig. 4E-F, *P* < 0.05), whereas increased in K562 cells with miR-17-3p knockdown (Fig. 4G-H, *P* < 0.05). The above results suggested that miR-17-3p could directly bind to BCL11A mRNA 3’-UTR and negatively regulate BCL11A expression.

### Effect of miR-17-3p on γ-globin expression in K562 cells

**Fig. 5:**
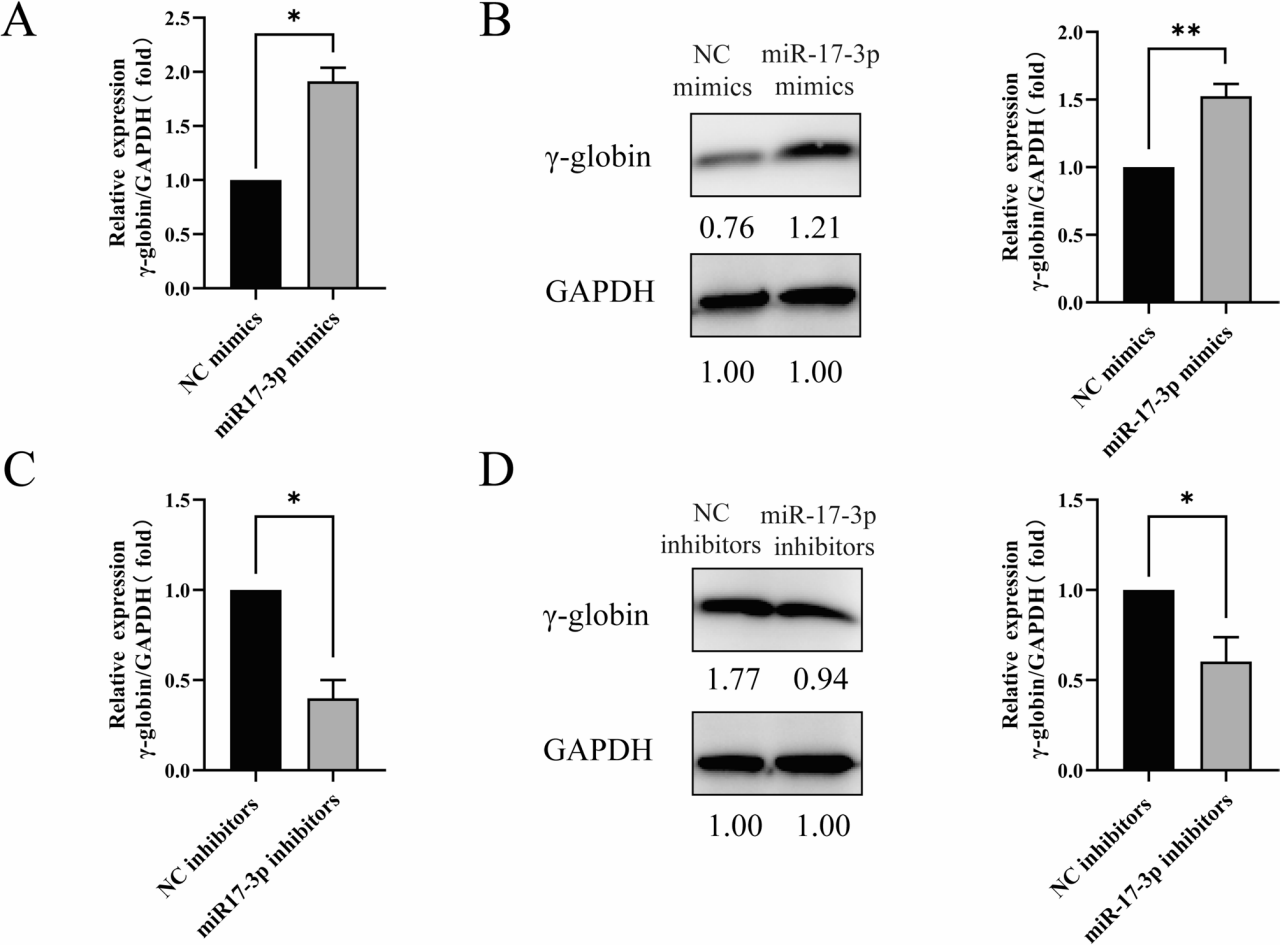
Effect of miR-17-3p on γ-globin expression of in K562 cells. Expression analysis of γ-globin mRNA (**A**) and protein (**B**) levels in the miR-17-3p mimics group and NC mimics group. Effect of knockdown of miR-17-3p on the expression analysis of γ-globin mRNA (**C**) and protein (**D**) levels. Error bars represented the means ± SD. *, *P* < 0.05; **, *P* < 0.01

The γ-globin gene reaction could present as a therapeutic target for β-thalassemia and might result from BCL11A downregulation. Therefore, we further detected γ-globin mRNA and protein expression in K562 cells transfected with miR-17-3p mimics or inhibitors to analyze the effect of miR-17-3p on γ-globin expression. Results of RT-qPCR and Western blotting analysis showed that, compared with the NC mimics group, the mRNA and protein expression levels of γ-globin were higher in K562 cells with miR-17-3p mimics (Fig. 5A-B, *P* < 0.05). However, the miR-17-3p inhibitors group showed a significant decrease in γ-globin mRNA and protein expression compared to the NC inhibitors group in K562 cells (Fig. 5C-D, *P* < 0.05). These results indicated that miR-17-3p reactivates the expression of γ-globin by targeting BCL11A.

### Effect of miR-17-3p on erythropoiesis of K562 cells

**Fig. 6:**
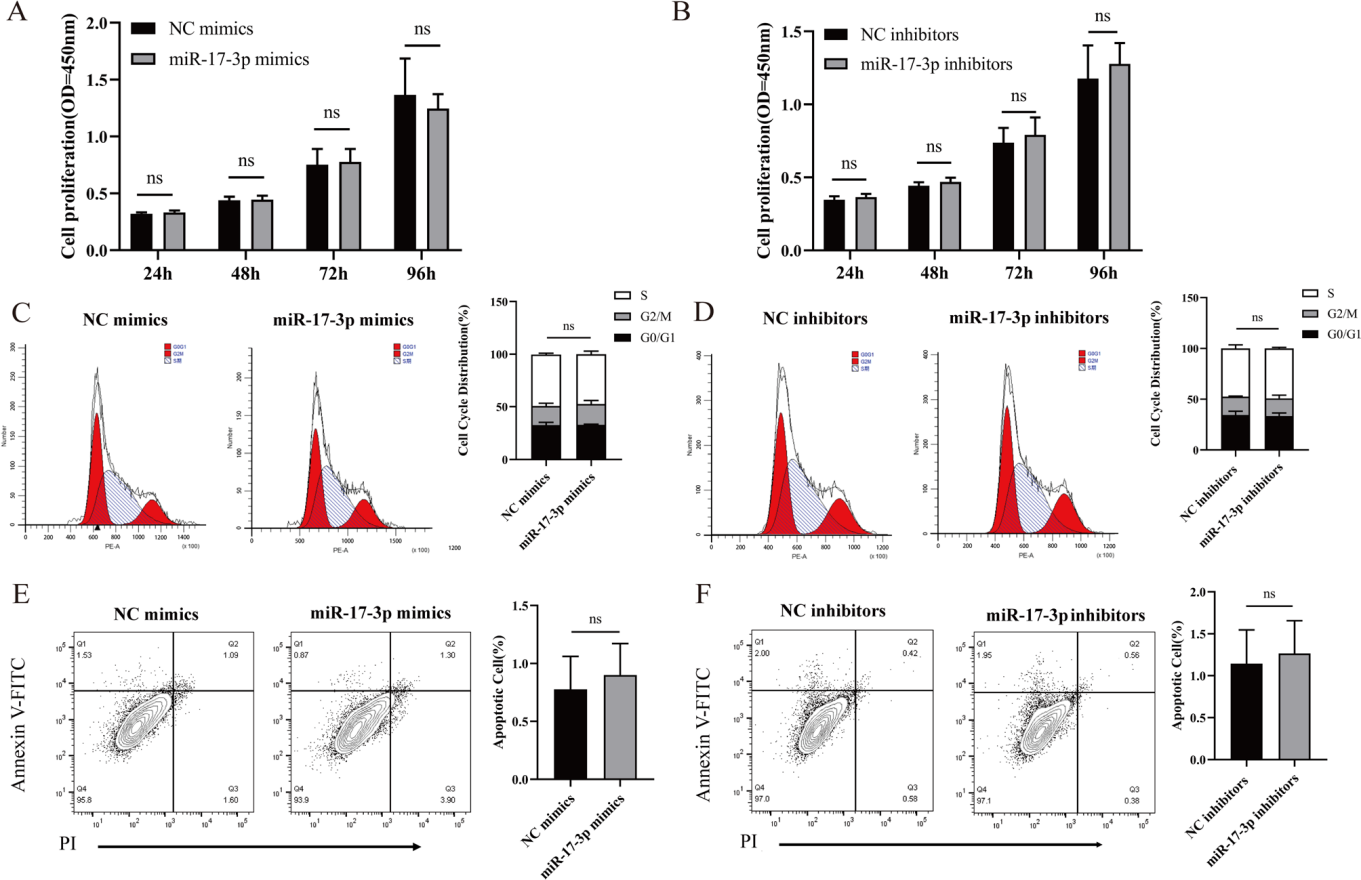
Effect of miR-17-3p overexpression or knockdown on the proliferation, cell cycle, and cell apoptosis of K562 cells. Changes in proliferation in the miR-17-3p mimics group (**A**), and the miR-17-3p inhibitors group (**B**). Effect of miR-17-3p overexpression (**C**), and miR-17-3p knockdown (**D**) on cell cycle. Effect of miR-17-3p overexpression (**E**), and miR-17-3p knockdown (**F**) on cell apoptosis. Error bars represented the means ± SD. ns, *P* > 0.05

To clarify the effect of miR-17-3p on erythropoiesis in β-thalassemia, we conducted the CCK-8 assay to detect the effect of miR-17-3p on cell proliferation in K562 cells. The results showed that the differences in absorbance values between the NC mimics group and the miR-17-3p mimics group at 24, 48, 72, and 96 h were not statistically significant (Fig. 6A, *P* > 0.05). Similarly, no significant differences were found in between the NC inhibitors group and the miR-17-3p inhibitors group (Fig. 6B, *P* > 0.05).

In order to assess the effect of miR-17-3p on the cell cycle, we used flow cytometry to detect the changes in the cell cycle 48 h after cell transfection in K562 cells. The results displayed that there were no significant differences in the G0/G1, S, and G2/M phase ratios between the NC mimics group and the miR-17-3p mimics group (Fig. 6C, *P* > 0.05). Similarly, no significant differences were observed in G0/G1, S, and G2/M phase ratios between the NC inhibitors group and the miR-17-3p inhibitors group (Fig. 6D, *P* > 0.05).

Subsequently, we detected the apoptosis rate of K562 cells after overexpression and knockdown of miR-17-3p. Results showed that miR-17-3p mimics group had no significant effect on apoptosis rate compared to the NC mimics group (Fig. 6E, *P* > 0.05). A similar finding on apoptosis rate was observed between the NC inhibitors group and the miR-17-3p inhibitors group (Fig. 6F, *P* > 0.05).

**Fig. 7:**
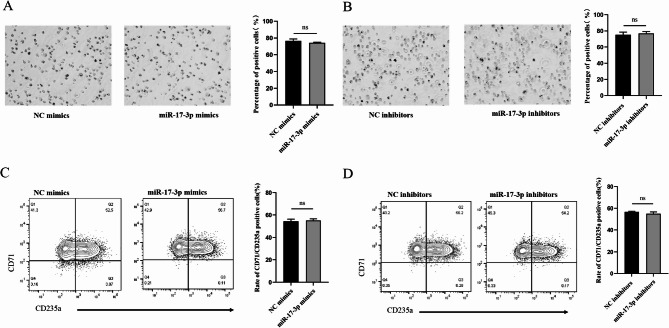
Effect of overexpression or knockdown of miR-17-3p on the erythroid differentiation of K562 cells. (**A**) Effect of miR-17-3p overexpression, and knockdown (**B**) on erythroid differentiation of K562 cells detected by benzidine blue staining. The effect of miR-17-3p overexpression (**C**), and knockdown (**D**) on erythroid differentiation of K562 cells detected by flow cytometry. Error bars represented the means ± SD. ns, *P* > 0.05

Moreover, Benzidine staining showed that no statistical significance was found in the rate of benzidine-positive staining cells between the NC mimics group and the miR-17-3p mimics group (Fig. 7A, *P* > 0.05). There were no statistically significant difference in the rate of benzidine-positive staining cells between the NC inhibitors group and the miR-17-3p inhibitors group (Fig. 7B, *P* > 0.05). Similarly, flow cytometry results showed that miR-17-3p mimics had no significant effect on the rate of CD71/CD235a positive cells compared to the NC mimics group (Fig. 7C, *P* > 0.05). miR-17-3p inhibitors had no significant effect on the rate of CD71/CD235a positive cells compared to the NC inhibitors group (Fig. 7D, *P* > 0.05). The above data demonstrated that miR-17-3p has no significant effect on cell proliferation, cell cycle, apoptosis and erythroid differentiation, implying that it may not affect erythropoiesis.

## Discussion

Reactivating the γ-globin gene is a promising therapeutic target for β-thalassemia, and increasing evidence shows that anemia symptoms in patients with β-thalassemia major can be ameliorated by inducing γ-globin expression and extending lifespan [18–19]. Previous studies have demonstrated the presence of multiple imbalances in miRNAs expression among β-thalassemia patients, with certain miRNAs exhibiting post-transcriptional silencing mediated by various transcription factors such as MYB, BCL11A, GATA1, and KLF1, which are implicated in the regulation of γ-globin gene expression [20–21]. Hence, the idea of reactivating γ-globin expression by regulating miRNA expression is worth pursuing and may be a new treatment for β-thalassemia.

In this study, we found that expression levels of miR-17-3p were significantly higher in patients with β-thalassemia, which was inconsistent with the downregulation of miR-17-3p expression in GSE241765 dataset. We hypothesized that the cause of this contradiction may be related to the different severities of β-thalassemia in the patients. HbF, composed of two γ-globin chains and two α-globin chains, serves as the predominant hemoglobin synthesized until birth and holds significant potential as a therapeutic approach for β-thalassemia. Here, we found that the expression levels of miR-17-3p in the patients with HbF ≥ 10 g/L were higher than those patients with HbF < 10 g/L. Moreover, miR-17-3p exhibited a robust positive correlation with the expression of HbF. Therefore, miR-17-3p holds great promise as a potential regulatory miRNA for modulating HbF level and represents an attractive therapeutic target for the treatment of β-thalassemia. In the correlation analysis of clinical indicators with miR-17-3p expression in patients with β-thalassemia, we found that the expression level of miR-17-3p was significantly negatively correlated with MCV, MCH, and HbA, but positively correlated with HbF, TBA, and GGT. It is hypothesized that the expression level of miR-17-3p might be associated with liver complications, hemolysis-related complications, and iron overload complications in patients with β-thalassemia.

miR-17-3p was suggested as a diagnostic biomarker in gastric [22], endometrial [23], colorectal [24], and breast cancer [25]. Furthermore, Shaker OG et al. discovered that miR-17-3p holds potential as a noninvasive biomarker for screening diabetic retinopathy (DR) and for the early diagnosis of proliferative diabetic retinopathy (PDR) [26]. Additionally, Bakhshi A et al. demonstrated that the expression level of miR-17-3p exhibits significant diagnostic potential for ST-segment elevation myocardial infarction (STEMI), and the combined assessment of miR-17-3p with NOD-like receptor thermal protein domain associated protein 3 (NLRP3) represents a promising novel diagnostic biomarker for STEMI [27]. Meanwhile, the results of ROC curve analysis showed that the expression of miR-17-3p in peripheral blood effectively discriminated β-thalassemia patients with different levels of HbF, indicating miR-17-3p might be used as a monitoring biomarker for this disease. In future study, we will continue to validate the performance of miR-17-3p as a monitoring biomarker for β-thalassemia in a large clinical sample, which will be helpful to predict changes in patients’ symptoms and plan appropriate treatment regimens.

Previous studies have primarily focused on miRNAs that target BCL11A and negatively regulate its expression, such as miR-210, which has been found to be associated with erythrocyte phenotype [28]. miR-210 regulates BCL11A gene expression by directly binding to the coding sequence of BCL11A mRNA, thus participating in the regulation of γ-globin genes [28]. The let-7 family of miRNAs has also been implicated in the regulation of BCL11A expression [29–32]. Lulli et al. [33] found that miR-486-3p negatively regulates BCL11A expression through direct binding to the BCL11A mRNA 3’-UTR, increasing γ-globin content and playing an important role in the hemoglobin conversion switch. Similarly, miR-30a [34] and miR-138-5p [35] can directly target BCL11A and upregulate γ-globin expression.

To verify the relationship between miR-17-3p and BCL11A, we initially validated that miR-17-3p primarily regulates gene expression in the cytoplasm by FISH assay. Also, we observed that miR-17-3p expression levels were significantly negatively correlated with BCL11A mRNA in β-thalassemia patients, and miR-17-3p might be associated with BCL11A and BCL11A was found to be a target of miR-17-3p by bioinformatic tools, which was verified by dual-luciferase reporter assay. Moreover, qRT-PCR and Western blotting experiments also demonstrated that miR-17-3p overexpression or knockdown could downregulate or upregulate BCL11A expression, respectively, with corresponding increases or decreases in γ-globin expression, which suggested that miR-17-3p reactivates the γ-globin expression by targeting BCL11A.

Yang et al. have reported [36] that miR-17-3p clusters are abundantly expressed in hematopoietic progenitor cells and correlated with hematopoietic cell expansion. The cell proliferation, cell cycle, apoptosis, and erythroid differentiation were investigated in K562 cells with miR-17-3p overexpression or knockdown to verify whether the effect of miR-17-3p on erythropoiesis. We found that miR-17-3p did not have a regulatory effect on cell proliferation, cell cycle, apoptosis, and erythroid lineage differentiation process in K562 cells. Previous studies have primarily focused on the tumorigenic role of miR-17-3p, demonstrating its impact on cell proliferation and migration in various tumors such as multiple myeloma, prostate cancer, and colon cancer [37–39], and also its effects on the growth and differentiation of cardiomyocytes [16]. However, in this study, miR-17-3p did not induce significant alterations in K562 cell proliferation, cycle, and apoptosis, which might be due to the different biological functions of miR-17-3p in different diseases, suggesting that there might be disease specificity in the biological functions of miR-17-3p.

However, these findings need to be further confirmed by large-scale clinical cases and animal models. For the clinical study of miR-17-3p in β-thalassemia, it is still necessary to incorporate large-scale clinical case analysis to verify the clinical significance of miR-17-3p in β-thalassemia. The use of the K562 cell line as a model for erythropoiesis has limitations due to its triploid karyotype and its classification as an erythroleukemia cell line. A cell model closer to human hemoglobin conversion studies and a humanized β-thalassemia mouse model will be selected to verify the mechanism of miR-17-3p in β-thalassemia both in vitro and in vivo, and to further analyze the importance of the miR-17-3p-BCL11A pathway, providing new insights into the target therapy of β-thalassemia.

## Conclusion

In this study, we found that upregulated miR-17-3p is associated with HbF in patients with β-thalassemia. miR-17-3p has no significant effect on cell proliferation, cell cycle, apoptosis or erythroid differentiation, implying that it may not affect erythropoiesis. miR-17-3p promotes γ-globin expression by targeting BCL11A, suggesting that miR-17-3p might be a promising target for inducing γ-globin reactivation in β-thalassemia treatment.

## Electronic supplementary material

Below is the link to the electronic supplementary material.

Supplementary Material 1: Figure

Supplementary Material 2: Tables

## Data Availability

Data sharing is not applicable to this article as no datasets were generated or analyzed during the current study.
