# Supplementary figures and images for: Upregulation of miR-17-3p is associated with HbF in patients with β-thalassemia and induces γ-globin expression by targeting BCL11A
Source: Orphanet J Rare Dis. 2025 May 30;20:260. doi: 10.1186/s13023-025-03806-0 (PMC12124070; doi:10.1186/s13023-025-03806-0)

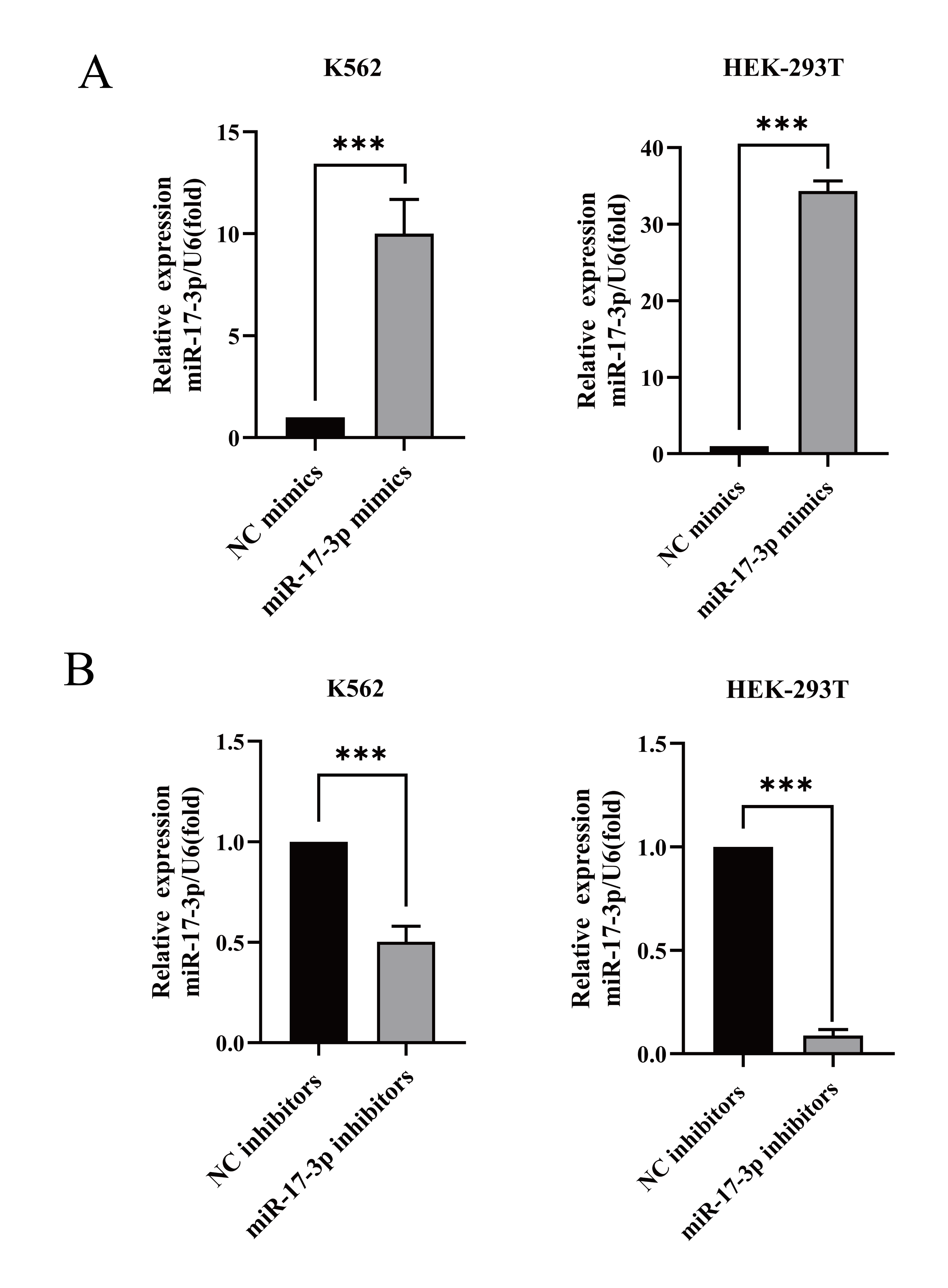

Supplement: Supplementary file 1 — Supplementary Material 1: Figure [file 13023_2025_3806_MOESM1_ESM.tif]
