# Supplementary material for: Upregulation of miR-17-3p is associated with HbF in patients with β-thalassemia and induces γ-globin expression by targeting BCL11A
Source: Orphanet J Rare Dis. 2025 May 30;20:260. doi: 10.1186/s13023-025-03806-0 (PMC12124070; doi:10.1186/s13023-025-03806-0)
Supplement: Supplementary file 2 — Supplementary Material 2: Tables [file 13023_2025_3806_MOESM2_ESM.docx]

**Supplementary Table 1.** The genotypes and clinical symptoms of patients with β-thalassemia intermedia and major

| Case no. | Sex | Age (year) | Genotypes | Clinical symptoms | | | |
| --- | --- | --- | --- | --- | --- | --- | --- |
|  |  |  |  | Anemia | hepatomegaly | splenomegaly | delayed growth and development |
| intermedia |  |  |  |  |  |  |  |
| 1# | male | 10 | β^CD17(A>T)^/β^CD26(G>A)^ | moderate | mild | N/A | no |
| 2# | female | 11 | β^IVS-II-654(C>T)^/β^-28(A>G)^ | moderate | mild | N/A | no |
| 3# | male | 9 | β^CD17(A>T)^/β^CD17(A>T)^ | moderate | N/A | N/A | no |
| 4# | male | 9 | β^CD17(A>T)^/β^CD17(A>T)^ | moderate | N/A | mild | no |
| 5# | female | 13 | β^CD41-42(-TCTT)^/β^-28(A>G)^ | moderate | mild | N/A | no |
| 6# | male | 9 | β^CD17(A>T)^/β^CD17(A>T)^ | moderate | N/A | N/A | no |
| 7# | male | 12 | β^IVS-II-654(C>T)^/β^-28(A>G)^ | moderate | mild | N/A | no |
| 8# | female | 9 | β^CD41-42(-TCTT)^/β^-28(A>G)^ | moderate | N/A | N/A | no |
| major |  |  |  |  |  |  |  |
| 9# | male | 5 | β^CD41-42(-TCTT)^/β^CD41-42(-TCTT)^ | severe | moderate | severe | yes |
| 10# | female | 7 | β^IVS-II-654(C>T)/^β^CD41-42(-TCTT)^ | severe | mild | moderate | yes |
| 11# | female | 10 | β^CD41-42(-TCTT)^/β^-28(A>G)^ | severe | moderate | severe | yes |
| 12# | male | 7 | β^CD41-42(-TCTT)^/β^IVS-I-1(G>T)^ | severe | severe | mild | yes |
| 13# | male | 8 | β^CD41-42(-TCTT)^/β^CD17(A>T)^ | severe | moderate | moderate | yes |
| 14# | female | 12 | β^CD17(A>T)^/β^CD17(A>T)^ | severe | moderate | mild | yes |
| 15# | male | 10 | β^CD41-42(-TCTT)^/β^CD41-42(-TCTT)^ | severe | mild | moderate | yes |
| 16# | male | 9 | β^CD41-42(-TCTT)^/β^CD17(A>T)^ | severe | severe | moderate | yes |
| 17# | female | 11 | β^CD41-42(-TCTT)^/β^CD41-42(-TCTT)^ | severe | moderate | moderate | yes |

N/A: not applicable.

**Supplementary Table 2.** Comparison of clinical indicators in patients with β-thalassemia and healthy controls

| Items | Healthy control (n=17) | β-thalassemia (n=17) | t/Z | *P* |
| --- | --- | --- | --- | --- |
| RBC (×10^12^/L) | 4.68±0.18 | 3.64±0.43 | -9.005 | <0.001 |
| Hb (g/L) | 132.00 (129.50,135.50) | 82.00 (63.50,85.50) | 4.981 | <0.001 |
| MCV (fL) | 83.34±2.24 | 77.69±6.40 | -3.43 | <0.001 |
| MCH (pg) | 28.43±0.80 | 24.72±2.19 | -6.58 | 0.001 |
| HbA (%) | 96.80 (96.55,97.25) | 81.88 (70.09,95.03) | 4.672 | <0.001 |
| HbA_2_ (%) | 2.80 (2.65,2.90) | 3.02 (2.51,3.25) | -1.881 | 0.052 |
| HbF (%) | 0.00 (0.00,0.40) | 7.39 (2.69,24.02) | -4.224 | <0.001 |
| PLT (×10^9^/L) | 280.76±64.92 | 423.82±195.28 | -2.866 | 0.012 |
| TBA (μmol/L) | 2.35±1.12 | 8.83±5.49 | -2.307 | 0.013 |
| TBIL (μmol/L) | 8.73±2.31 | 26.90±8.60 | -7.659 | 0.001 |
| DBIL (μmol/L) | 2.00 (1.6, 2.2) | 8.80 (6.69,11.6) | -7.743 | <0.001 |
| ALT (U/L) | 11.85 (10.94,15.53) | 26.00 (20.80,44.00) | -3.029 | 0.001 |
| AST (U/L) | 24.60 (22.98,26.68) | 30.00 (25.00,40.00) | -2.680 | 0.007 |
| ALP (U/L) | 287.80 (242.30,300.63) | 205.00 (176.00,291.00) | 0.262 | 0.101 |
| GGT (U/L) | 12.80 (11.83,14.33) | 13.00 (11.00,14.00) | 0.574 | 0.812 |
| TP (g/L) | 72.10 (71.30,74.50) | 67.50 (63.30,72.70) | 2.908 | 0.002 |
| ALB (g/L) | 45.55 (44.03,46.18) | 45.00 (43.90,46.70) | 0.174 | 0.957 |
| GLOB (g/L) | 27.67±2.74 | 23.60±4.03 | 3.212 | 0.125 |
| PA (g/L) | 230.83±2.74 | 198.79±43.14 | 1.447 | 0.022 |
| CHE (U/L) | 9155.50 (8314.25,10084.25) | 6192.00 (5323.00,7340.00) | -2.240 | 0.025 |
| SF (μg/L) | 52.94±20.92 | 2861.87±1077.71 | -5.728 | 0.003 |
